# Combination drug development in BRAF mutant colorectal cancer

**DOI:** 10.18632/oncoscience.399

**Published:** 2018-04-29

**Authors:** Michael Lam, Shubham Pant, Timothy A. Yap

**Affiliations:** Department of Investigational Cancer Therapeutics (Phase I Clinical Trials Program), and the Department of Thoracic/Head and Neck Medical Oncology; Khalifa Institute for Personalized Cancer Therapy;The Institute for Applied Cancer Science, The University of Texas MD Anderson Cancer Center, 1515 Holcombe Boulevard, Unit 455, Houston, Texas 77030, USA

**Keywords:** BRAF mutant colorectal cancer, resistance, MEK, ERK, combinations

Despite recent therapeutic advances, the management of patients with BRAF V600E mutant colorectal cancer (bmCRC) remains an area of clinical need. It is associated with a unique clinical phenotype, including its proximal tumor location, poorly differentiated histology, as well as peritoneal and nodal spread. Chemoresistance is often attributed to bmCRC, although this does not always manifest in progression- free survival (PFS) differences compared with its wild- type counterparts. However, overall survival (OS) remains universally poor irrespective of the therapy [1]. Furthermore, molecularly targeted therapy approaches to improve survival outcomes in these patients with BRAF inhibitors have been disappointing relative to the impressive responses observed in melanoma. In selected molecular basket clinical trials, the overall response rate (ORR) to these strategies in bmCRC was 0-5% and median progression free survival (PFS) was 2.1 months [2].

The preclinical findings demonstrating that BRAF inhibition releases negative feedback on epidermal growth factor receptor (EGFR) signalling in CRC, but not melanoma, have led to the contemporary combination strategies utilized today [3]. Targeting BRAF and EGFR concurrently abrogates the intrinsic mechanism of resistance in bmCRC, leading to modest improvements in response rates over single agent therapy. Chemotherapy has also been utilized to improve efficacy. For example, the SWOG1406 phase 2 study comparing irinotecan and cetuximab with or without vemurafenib demonstrated that the addition of a BRAF inhibitor resulted in an improved median PFS (4.4 vs 2.0 months, HR 0.42 [95% CI, 0.26- 0.66]; p < 0.001), and is a promising strategy for the treatment of bmCRC [4]. Partial responses (PR) were observed in 16% vs 4.0%, while disease stabilization (SD) was seen in 50% vs 17% in the experimental arm compared to the control arm, respectively. Despite these incremental gains made with such combination approaches, the absolute benefit remains modest.

**Figure 1:**
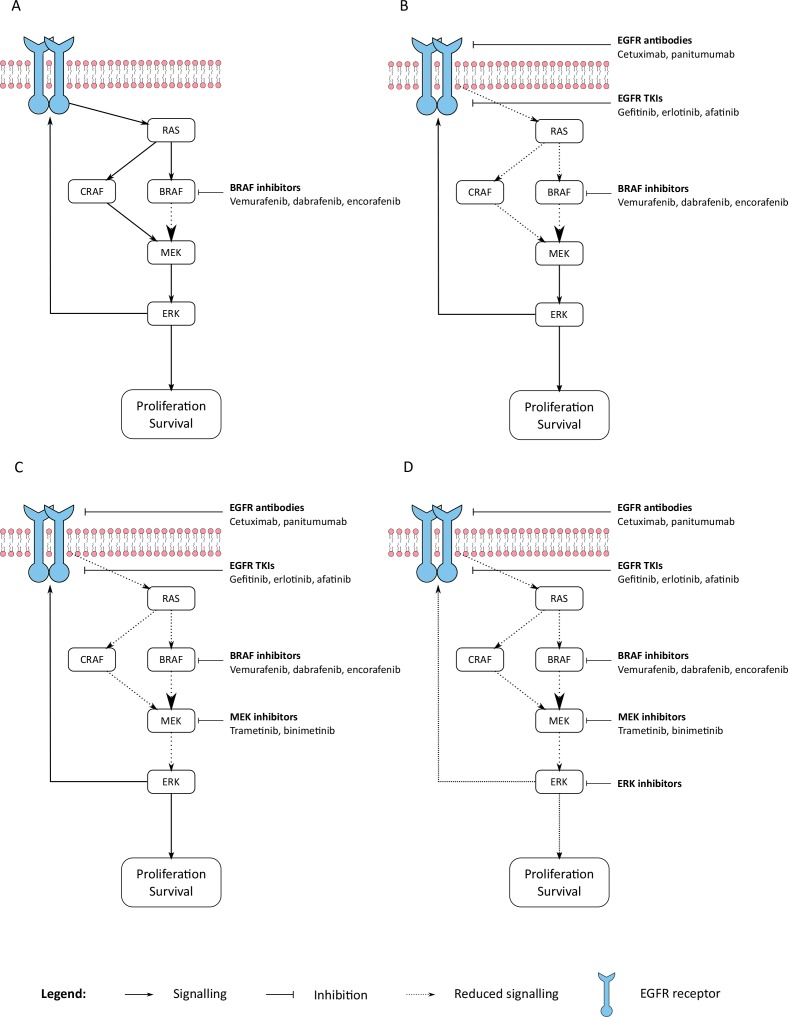
Targeted strategies for BRAFV600E mCRC (**A**) Single agent BRAF inhibition resulting in EGFR EGFR reactivation. (**B**) Dual targeting with BRAF inhibition and EGFR addressing intrinsic resistance in mCRC. (**C**) Additional MEK inhibition to current BRAF + EGFR backbones. (**D**) Addition of ERK inhibition has shown activity in preclinical models that have developed resistance to current targeted combinations. mCRC - metastatic colorectal cancer; EGFR - epidermal growth factor receptor.

Future combination studies are aimed towards strategies that further inhibit MAPK activity or which target hypothesized resistance pathways (Figure 1). The addition of a MEK inhibitor is being assessed in the phase III BEACON study (NCT02928224), where encorafenib (BRAF inhibitor), binimetinib (MEK inhibitor) and cetuximab are being compared to encorafenib plus cetuximab, or irinotecan (or FOLFIRI) and cetuximab. Preclinical models that developed resistance to BRAF and EGFR inhibitor combinations displayed sensitivity with the addition of ERK inhibitors to such doublet combinations [5]. The incorporation of ERK inhibitors with BRAF and EGFR doublet combinations appears to be a promising approach for circumventing resistance and improving efficacy in bmCRC.

Despite the clear rationale and need for combinations to minimize drug resistance and issues of intratumor heterogeneity, the temptation to add more targeted treatments to combination regimens needs careful consideration. True synergism needs to be demonstrated in order to justify the addition of novel therapies to existing doublet combinations in view of the potential added toxicities that will narrow the therapeutic window. The combination of small molecule inhibitors often requires the modulation of dose and/or schedule of the monotherapy maximum tolerated doses previously established for each agent, which may ultimately compromise target and pathway inhibition, and affect combination synergy [6]. For example, in the phase 1b trial combining cetuximab, encorafenib with or without the PI3Kα isoform-specific inhibitor alpelisib, antitumor efficacy was not improved with the triplet combination and was instead associated with greater predicted toxicities of nausea, diarrhea and hyperglycemia versus the doublet arm [7]. In addition, while many preclinical studies report the upregulation of the PI3K/AKT pathway after BRAF inhibition in bmCRC, the concurrent inhibition of both signaling pathways has not led to definitive gains in efficacy in the clinic [5]. Measured and thoughtful assessments to carefully balance combination-associated toxicities and efficacy will therefore be important before committing more patients to larger trials of any combinatorial regimen.

It is clear that some patients demonstrate tumor regression and sustain durable responses, while others develop disease progression quickly. Further advances in the management of bmCRC will come from improving our understanding of the underlying mechanisms of both intrinsic and acquired resistance to different therapies. Recent transcriptomic analyses of bmCRC revealed two distinct subtypes, BM1 and BM2, revealing tumor heterogeneity at a gene expression level, which is not accounted for by the presence of microsatellite instability (MSI) status [8]. Occurring in an approximate 1:2 proportion, these subtypes have unique biological characteristics; BM1 demonstrates KRAS/AKT pathway upregulation, epithelial mesenchymal transition processes and immune activation, whereas BM2 is deregulated for cell cycle processes. These distinct subtypes provide an opportunity in future to be more defined with regards to developing combination strategies for specific patient populations with bmCRC. However, further analytical validation is still required before large scale gene expression profiling can be robustly utilized for patient stratification.

Understanding and tackling acquired resistance in treating bmCRC appears potentially achievable in the near future, with key components along the MAPK pathway, such as EGFR, RAS, RAF and MEK, already under active research in preclinical models and in the clinic [5]. Given the challenges associated with obtaining tumor biopsies at disease progression, the emergence of circulating tumor DNA (ctDNA) in tracking mutant allele clones may aid in better understanding such resistance mechanisms to current targeted combinations in bmCRC.
